# Supplementary material for: Resolving the full spectrum of human genome variation using Linked-Reads
Source: Genome Res. 2019 Apr;29(4):635–45. doi: 10.1101/gr.234443.118 (PMC6442396; doi:10.1101/gr.234443.118)
Supplement: Supplemental Material [file supp_gr.234443.118_Supplemental_longranger-2.2.zip › longranger-2.2.tar.bz2!/lr2.2-public/lib/stan/cmdstan/stan/doc/api/html/rev_2numeric__limits_8hpp_source.html]

Stan: src/stan/agrad/rev/numeric\_limits.hpp Source File


|  |  |
| --- | --- |
|  | Stan  2.5.0  probability, sampling & optimization |


- Main Page
- Related Pages
- Namespaces
- Classes
- Files

- File List
- File Members

All Classes Namespaces Files Functions Variables Typedefs Enumerations Enumerator Friends Macros Pages

- src
- stan
- agrad
- rev

numeric\_limits.hpp

Go to the documentation of this file.

1 #ifndef STAN\_\_AGRAD\_\_REV\_\_NUMERIC\_LIMITS\_HPP

2 #define STAN\_\_AGRAD\_\_REV\_\_NUMERIC\_LIMITS\_HPP

3

4 #include <limits>

5 #include <stan/agrad/rev/var.hpp>

6

7 namespace std {

8

15  template<>

16  struct numeric\_limits<stan::agrad::var> {

17  static const bool is\_specialized = true;

18  static stan::agrad::var min() { return numeric\_limits<double>::min(); }

19  static stan::agrad::var max() { return numeric\_limits<double>::max(); }

20  static const int digits = numeric\_limits<double>::digits;

21  static const int digits10 = numeric\_limits<double>::digits10;

22  static const bool is\_signed = numeric\_limits<double>::is\_signed;

23  static const bool is\_integer = numeric\_limits<double>::is\_integer;

24  static const bool is\_exact = numeric\_limits<double>::is\_exact;

25  static const int radix = numeric\_limits<double>::radix;

26  static stan::agrad::var epsilon() { return numeric\_limits<double>::epsilon(); }

27  static stan::agrad::var round\_error() { return numeric\_limits<double>::round\_error(); }

28

29  static const int min\_exponent = numeric\_limits<double>::min\_exponent;

30  static const int min\_exponent10 = numeric\_limits<double>::min\_exponent10;

31  static const int max\_exponent = numeric\_limits<double>::max\_exponent;

32  static const int max\_exponent10 = numeric\_limits<double>::max\_exponent10;

33

34  static const bool has\_infinity = numeric\_limits<double>::has\_infinity;

35  static const bool has\_quiet\_NaN = numeric\_limits<double>::has\_quiet\_NaN;

36  static const bool has\_signaling\_NaN = numeric\_limits<double>::has\_signaling\_NaN;

37  static const float\_denorm\_style has\_denorm = numeric\_limits<double>::has\_denorm;

38  static const bool has\_denorm\_loss = numeric\_limits<double>::has\_denorm\_loss;

39  static stan::agrad::var infinity() { return numeric\_limits<double>::infinity(); }

40  static stan::agrad::var quiet\_NaN() { return numeric\_limits<double>::quiet\_NaN(); }

41  static stan::agrad::var signaling\_NaN() { return numeric\_limits<double>::signaling\_NaN(); }

42  static stan::agrad::var denorm\_min() { return numeric\_limits<double>::denorm\_min(); }

43

44  static const bool is\_iec559 = numeric\_limits<double>::is\_iec559;

45  static const bool is\_bounded = numeric\_limits<double>::is\_bounded;

46  static const bool is\_modulo = numeric\_limits<double>::is\_modulo;

47

48  static const bool traps = numeric\_limits<double>::traps;

49  static const bool tinyness\_before = numeric\_limits<double>::tinyness\_before;

50  static const float\_round\_style round\_style = numeric\_limits<double>::round\_style;

51  };

52

62  inline int isnan(const stan::agrad::var& a) {

63  return isnan(a.val());

64  }

65

75  inline int isinf(const stan::agrad::var& a) {

76  return isinf(a.val());

77  }

78

79 }

80 #endif

std::numeric\_limits< stan::agrad::var >::quiet\_NaN

static stan::agrad::var quiet\_NaN()

**Definition:** numeric\_limits.hpp:40

std::numeric\_limits< stan::agrad::var >::denorm\_min

static stan::agrad::var denorm\_min()

**Definition:** numeric\_limits.hpp:42

stan::agrad::var::val

double val() const

Return the value of this variable.

**Definition:** var.hpp:209

stan::math::max

double max(const double a, const double b)

**Definition:** max.hpp:7

std::numeric\_limits< stan::agrad::var >::round\_error

static stan::agrad::var round\_error()

**Definition:** numeric\_limits.hpp:27

std::numeric\_limits< stan::agrad::var >::signaling\_NaN

static stan::agrad::var signaling\_NaN()

**Definition:** numeric\_limits.hpp:41

std::numeric\_limits< stan::agrad::var >::max

static stan::agrad::var max()

**Definition:** numeric\_limits.hpp:19

std::numeric\_limits< stan::agrad::var >::infinity

static stan::agrad::var infinity()

**Definition:** numeric\_limits.hpp:39

std::numeric\_limits< stan::agrad::var >::min

static stan::agrad::var min()

**Definition:** numeric\_limits.hpp:18

var.hpp

stan::agrad::var

Independent (input) and dependent (output) variables for gradients.

**Definition:** var.hpp:27

std::isinf

int isinf(const stan::agrad::var &a)

Checks if the given number is infinite.

**Definition:** numeric\_limits.hpp:75

std::isnan

int isnan(const stan::agrad::var &a)

Checks if the given number is NaN.

**Definition:** numeric\_limits.hpp:62

std::numeric\_limits< stan::agrad::var >::epsilon

static stan::agrad::var epsilon()

**Definition:** numeric\_limits.hpp:26

stan::math::min

double min(const double a, const double b)

**Definition:** min.hpp:7


---

[ Stan Home Page ]

*© 2011–2014,
Stan Development Team.*
